# *Flavobacterium psychrophilum* as an Important Pathogen Associated with Overwintering Mortality Syndrome in Grass Carp (*Ctenopharyngodon idella*): Isolation, Characterization, and Pathogenicity

**DOI:** 10.3390/ani16101465

**Published:** 2026-05-10

**Authors:** Hongyang Song, Mingyang Xue, Yong Zhou, Wenzhi Liu, Wei Liu, Nan Jiang, Yiqun Li, Yan Meng, Xin Ren, Yuding Fan

**Affiliations:** 1 Yangtze River Fisheries Research Institute, Chinese Academy of Fishery Sciences, Wuhan 430223, China; 2 College of Fisheries and Life Science, Shanghai Ocean University, Shanghai 201306, China

**Keywords:** grass carp (*Ctenopharyngodon idella*), *Flavobacterium psychrophilum*, overwintering mortality syndrome, pathogenicity, antimicrobial resistance

## Abstract

Grass carp (*Ctenopharyngodon idella*), a key species in China’s freshwater aquaculture, faces hindered industrial development due to overwintering mortality disease outbreaks. A Gram-negative bacterial strain, designated Hbh25210, was isolated from overwintering mortality-diseased grass carp in a Hubei Province farm. It was identified as *Flavobacterium psychrophilum* by 16S rRNA gene sequencing, showing 100% homology to reference strains in GenBank. The strain can be successfully cultured at 15 °C within 3 to 5 days. Multilocus sequence typing (MLST) classified it as ST-51 (CC-ST56), while serotyping assigned it to the 0 type, indicating phylogenetic affinity to Japanese cyprinid isolates. Artificial infection experiments confirmed its pathogenicity: muscle injection resulted in 15–75% mortality in grass carp, with clinical symptoms consistent with natural infections. Histopathological changes included hepatic necrosis, splenic congestion, glomerular swelling, and muscle fiber dissolution; intestinal lesions were particularly prominent in infected fish. 16S rRNA gene sequencing of diseased tissues showed *Flavobacterium psychrophilum* constituted over 60% of the microbial community in most ulcerative lesions samples and reached as high as 98.8% in certain cases. Strain Hbh25210 exhibited high-level resistance to multiple antibiotics, including ciprofloxacin, cefadroxil, neomycin, florfenicol, sulfamethoxazole, doxycycline, trimethoprim–sulfamethoxazole, and flumequine. Conversely, it was relatively sensitive to enrofloxacin, gentamicin, and amikacin. Quantitative PCR showed the bacterial load in muscle remained the highest, followed by the intestine and spleen, which peaked at 96 h post-injection. In conclusion, this study indicates that *Flavobacterium psychrophilum* is an important pathogen associated with overwintering mortality syndrome in grass carp. Our research findings can provide a basis for the effective prevention and control of overwintering mortality syndrome in major freshwater fish species.

## Simple Summary

Grass carp is a major freshwater farmed fish in China. Overwintering mortality syndrome has inflicted severe economic losses on the aquaculture industry, with its main causative agent remaining unclear. This study isolated and identified a highly pathogenic *Flavobacterium psychrophilum* strain Hbh25210 from diseased grass carp during overwintering in Hubei Province. Experiments verified that the strain causes 15–75% mortality in grass carp and accumulates heavily in muscle, the spleen and the intestine. We also characterized its biological and antimicrobial resistance profiles, supporting its important role in the syndrome. These findings provide practical references for disease diagnosis and help reduce aquaculture losses for farmers.

## 1. Introduction

As one of the most economically important aquaculture species globally, grass carp (*Ctenopharyngodon idella*) holds a dominant position in China’s freshwater aquaculture industry [1]. According to the China Fisheries Statistical Yearbook 2025, the national output of freshwater-cultured grass carp reached 6.1649 million tons in 2024, with Hubei Province ranking second nationally at 835,075 tons. However, the expansion of intensive farming has intensified disease challenges. Key pathologies include viral hemorrhagic disease caused by grass carp reovirus (GCRV), which is associated with a mortality rate exceeding 85% [2], and bacterial infections—including enteritis, ulcerative syndrome, and septicemia—triggered by *Aeromonas hydrophila* and *Aeromonas veronii* [3,4]. Notably, since 2019, overwintering-associated mortality has emerged as a critical disease threat, characterized by persistent and progressive mortality in grass carp populations. Grass carp overwintering mortality syndrome has caused severe economic losses to the aquaculture industry. As the disease is driven by complex multifactorial interactions, its etiology has long remained elusive. Therefore, it is urgent to identify the causative pathogen and clarify its pathogenic characteristics.

This overwintering mortality phenomenon is not restricted to grass carp. Since 2019, outbreaks of persistent progressive mortality have been extensively documented across multiple freshwater fish species in various regions of China following the overwintering period. Affected grass carp exhibit systemic congestion, caudal peduncle ulceration, and profuse hemorrhage from ulcerated areas; bighead carp develop caudal peduncle white scabs accompanied by systemic congestion, scale loss, fin base inflammation, and mouth ulcers; crucian carp show dermal hemorrhage, caudal erosion, exophthalmos, ascites, and intestinal fragility [5]. Domestic aquatic researchers are accustomed to referring to the disease that causes the death of major commercial freshwater fish during overwintering as “overwintering mortality syndrome”. The increasing frequency of overwintering mortality syndrome is associated with recurrent extreme winter climatic conditions and nation-wide trade in aquatic products, which accelerate the cross-regional spread of opportunistic pathogens [6]. These microorganisms compromise the dermal barrier of grass carp by inducing epidermal ulceration, thereby impairing mucosal immune defenses. This cascade facilitates the proliferation of cold-adapted microbes such as *Shewanella putrefaciens*, enhances muscle amino acid catabolism, and disrupts the aquatic microecological balance—ultimately resulting in mortality [7,8].

*Flavobacterium psychrophilum*, a Gram-negative bacterium belonging to the phylum Bacteroidetes first isolated from *Oncorhynchus kisutch* in 1948 [9], is particularly relevant in this context. This pathogen infects diverse fish species, including salmonids, roach, and goldfish [5,10,11,12,13]. External lesions in affected fish are typically evident, generally presenting as cutaneous ulceration, scale exfoliation, muscular hemorrhage at the caudal peduncle, and associated tissue necrosis [14]. Disease onset typically occurs at water temperatures of 4–15 °C, with severe manifestations below 10 °C [15]. Multiple putative virulence factors have been identified in *Flavobacterium psychrophilum*, primarily extracellular proteases that degrade extracellular matrix components such as elastin, fibrinogen, type IV collagen, actin, and myosin [16,17,18]. However, there are few detailed reports on *Flavobacterium psychrophilum* and its virulence factors in relation to the overwintering mortality syndrome of grass carp.

This study aimed to identify the causative pathogen associated with mass mortality in farmed grass carp following overwintering at an aquaculture facility in Honghu City, Hubei Province, in mid-February 2025. Using 16S rRNA gene sequencing, we isolated and identified the dominant pathogen and characterized its physiological and biochemical properties, conducted molecular typing, and assessed its pathogenicity. Experimental infection via intramuscular injection was performed to quantify bacterial loads in tissues. The overall objective was to clarify the primary etiological agent and pathogenic mechanism of grass carp overwintering mortality syndrome, thereby supporting the development of effective prevention and control strategies for this disease.

## 2. Materials and Methods

### 2.1. Experimental Materials

Healthy grass carp came from the original seed farm of four major Chinese carp in the old river of Shishou (Hubei, China). The body length of grass carp is 10 ± 2 cm, and the body weight is about 25 ± 5 g. All fish were temporarily reared in aquaculture water at 15 °C before the experiment and were fed twice a day at 2% body weight. Prior to experimental use, all individuals were confirmed negative for viral and major bacterial pathogens via RT-PCR and standard microbiological culture. The diseased fish were collected from a farmer in Honghu City, Hubei Province. Relevant breeding information and clinical symptoms were recorded, and wound tissues as well as immune tissues were collected for analysis.

### 2.2. Multi-Tissue 16S rDNA Sequencing

Liver and wound tissue samples (0.2 g each) were collected from moribund fish, with three biological replicates per tissue type. Samples were immediately frozen in liquid nitrogen and sent to Wuhan Tianyi Huayu Gene Technology Co., Ltd. (Wuhan, China) for sequencing. Total bacterial DNA was extracted using a bacterial DNA kit (Tiangen, Beijing, China). The V3–V4 highly variable region of the 16S rDNA gene (341F and 806R) was amplified using the total DNA as a template (Table 1). After purification and recovery of PCR products, sequencing libraries were constructed and sequenced on the NovaSeq platform (Illumina Inc., San Diego, CA, USA) [1]. After quality control, ASVs/OTUs were obtained, followed by taxonomic annotation, principal coordinate analysis (PCoA), α-diversity analysis, and β-diversity analysis [2]. Dominant pathogenic bacteria were isolated, cultured, and purified based on the sequencing results.

### 2.3. Bacteria Isolation and Identification

Under sterile conditions, ulcerative lesions on the body surface, as well as liver, spleen, and kidney tissues, were streaked onto TYES solid medium (TopBio, Qingdao, China) and incubated at 15 °C for 96 h. The plates inoculated with liver, spleen, and kidney tissues showed complex and diverse bacterial communities without any distinct dominant colony. In contrast, prominent yellow, smooth, and translucent colonies were observed on the plates from ulcerative lesion samples, which were picked for identification. Genomic DNA was extracted using a bacterial genomic DNA extraction kit. 16S rDNA was amplified by PCR with primers 27F and 1492R (Table 1). Amplification products were detected by 1.5% agarose gel electrophoresis and then sent to Wuhan Tianyi Huayu Gene Technology Co., Ltd. (Wuhan, China) for sequencing. Sequencing results were subjected to BLAST alignment in the NCBI database (National Center for Biotechnology Information). Several strains with high homology were selected for multiple sequence alignment and phylogenetic tree construction using the neighbor-joining method in MEGA 7.0 software. The strain was preliminarily identified as *Flavobacterium psychrophilum* based on 16S rRNA sequences. After sequencing verification, the strain was stored in 50% glycerol at −80 °C and designated as Hbh25210.

### 2.4. Morphological Observation and Physicochemical and Biochemical Properties of the Strain

*Flavobacterium psychrophilum* was spread onto glass slides, heat-fixed, and subjected to Gram staining (Biosharp, Beijing, China). Bacterial morphology and staining characteristics were observed under an oil immersion lens (Olympus, Tokyo Metropolis, Japan) [6]. The strain was fixed in 2.5% glutaraldehyde solution (Yuanye, Shanghai, China), dehydrated, dried, sputter-coated with gold, and then observed for colony morphology under a scanning electron microscope (HITACHI, Tokyo Metropolis, Japan) [7]. Physiological and biochemical identification was carried out according to the instructions of the bacterial microbiochemical identification tube (Hopebio, Qingdao, China).

Multilocus sequence typing (MLST) and serotype analysis were performed using the genomic DNA of the strain as the template. Seven pairs of housekeeping genes (*trpB*, *gyrB*, *dnaK*, *fumC*, *murG*, *tuf*, *atpA*) and primers, as well as the corresponding PCR protocol, were obtained through the MLST website (Table 1). According to the method reported by Rochat et al. [5], four pairs of primers were used for serotype identification of the strain by multiplex PCR (mPCR) (Table 1). PCR products were analyzed by 1.5% agarose gel electrophoresis, and serotypes were determined based on the amplified product sizes. The amplified bands corresponding to serotypes 0, 1, 2 and 3 were 188 bp, 188 bp + 549 bp, 188 bp + 841 bp and 188 bp + 361 bp, respectively.

### 2.5. Drug Sensitivity Testing

Under aseptic conditions, 100 μL of the enriched bacterial suspension was spread onto TYES agar medium. Twelve antimicrobial susceptibility discs were placed on the medium surface, followed by incubation at 15 °C for 96 h. The diameter of the inhibition zone was measured using a Vernier caliper, and values were recorded for each antimicrobial agent (no technical replicates or quality control strains included).

### 2.6. Artificial Infection Experiment and Histopathological Observations

Healthy grass carp temporarily cultured for 15 days were randomly divided into 4 groups, with 20 grass carp per group. The Hbh25210 strain was inoculated in TYES liquid medium and shaken at 15 °C and 150 r/min for 72 h. After centrifugation, Phosphate-Buffered Saline (Cytiva, Shenzhen, China) was rinsed three times to prepare bacterial suspension [8]. The water temperature was maintained at 15 °C during the experiment, and each group was intramuscularly injected with 100 μL of 2.00 × 10^8^, 2.00 × 10^7^, and 2.00 × 10^6^ colony-forming units (CFU)/mL, respectively. The control group was injected with the same volume of PBS. The death of grass carp within 15 days was recorded and observed, and survival curves were constructed using the Kaplan–Meier (KM) method with the log-rank test. Three moribund or dead grass carp from each group were dissected after anesthesia. Tissue samples, including ulcerated skin lesions, liver, spleen, and kidney, were streaked directly onto TYES solid medium for the isolation and identification of pathogenic bacteria.

Liver, spleen, kidney, intestine, and muscle tissues were collected from both farmed fish and experimentally infected fish at 72 h post-injection (hpi). Samples were fixed in 4% paraformaldehyde (Biosharp, Beijing, China); processed through dehydration, embedding, sectioning, and hematoxylin–eosin (HE) staining; and examined under an optical microscope for histopathological analysis [9].

### 2.7. Quantitative Detection of Flavobacterium psychrophilum in Various Tissues

Bacterial pellets were collected by centrifugation for genomic DNA extraction. A 164 bp fragment of the *rpoC* gene was amplified by PCR (Table 2) and quantified using a Nanodrop™ 2000 spectrophotometer (ThermoFisher, Waltham, MA, USA). Target copy numbers were calculated based on a single fragment mass of 1.797 × 10^−7^ pg [10,11]. Serial 10-fold dilutions of purified DNA were used to construct a TaqMan qPCR (Quantitative Real-time polymerase chain reaction) standard curve ranging from 2.3 × 10^9^ to 2.3 × 10^2^ copies/μL (Table 2). qPCR was performed in a 20 μL mixture containing 10 μL of 2× Hieff Unicon^®^ TaqMan Probe Master Mix (Arcegen Inc., Gaithersburg, MD, USA), 0.4 μL each of forward and reverse primers (10 μM), 0.4 μL of TaqMan probe (10 μM), 1 μL of template DNA, and 7.8 μL of ddH_2_O; cycling conditions were 95 °C for 10 min, 40 cycles (95 °C 15 s, 60 °C 1 min, 72 °C 1 s), and 40 °C for 10 s [12].

Fifteen healthy grass carp were intramuscularly injected with 100 μL of *Flavobacterium psychrophilum* suspension at 2 × 10^8^ CFU/mL. Liver, spleen, kidney, intestine, gill, heart, brain and muscle were collected at 24, 48 and 96 hpi for bacterial load analysis. Three grass carp were sampled at each infection time point. Bacterial genomic DNA was extracted from 20 mg of tissue samples and detected using the aforementioned qPCR method with negative controls. Bacterial loads were calculated based on Ct values and the standard curve.

### 2.8. Statistical Analysis

Statistical analyses were performed using GraphPad Prism 10 with two-way ANOVA followed by Duncan’s multiple range test for post hoc comparisons, and survival curves were analyzed by the Kaplan–Meier method with the log-rank test. Quantitative data are expressed as mean ± SD ($\overset{-}{x}$ ± SD), and statistical significance was defined at *p* < 0.001.

## 3. Results

### 3.1. Clinical Symptoms of Diseased Fish

**Figure 1:**
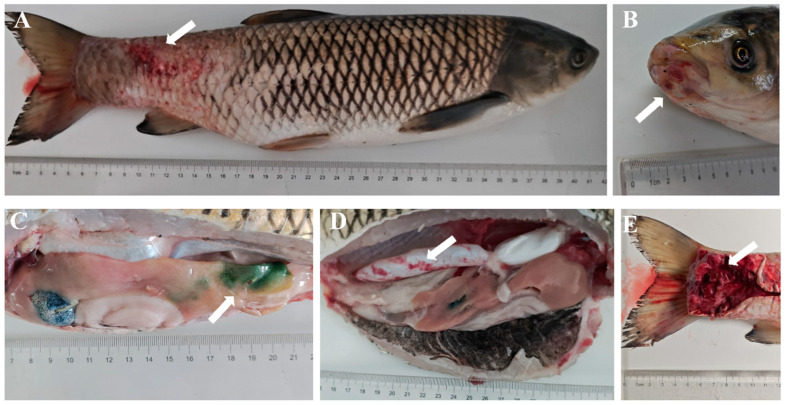
Clinical symptoms of diseased grass carp in Honghu City. (**A**) Muscle enlargement and hemorrhage at the caudal peduncle (white arrow); (**B**) Snout ulceration and hyperemia (white arrows); (**C**) Greenish discoloration of the liver without gallbladder rupture (white arrow); (**D**) Hyperemia at the distal end of the swim bladder surface (white arrow); (**E**) Dissection of the caudal peduncle reveals muscle tissue dissolution and blood vessel rupture (white arrow).

The diseased grass carp measured an average length of 40 ± 5 cm and an average weight of 700 ± 100 g; the water temperature was 8 ± 3 °C. The disease occurred on a fish farm with a total of four ponds, with an overall mortality rate of approximately 40%, and the number of affected fish was roughly estimated at 3200. Key clinical symptoms included abnormal swimming, scale shedding with varying degrees of skin hemorrhage, and snout ulceration. In some individuals, localized swellings appeared on the dorsal fin and caudal skin; dissection revealed bloody, torn tissue with profuse bleeding at these swollen sites. Additionally, a subset of diseased fish exhibited greenish discoloration of the liver without gallbladder rupture, alongside congestive lesions in the swim bladder (Figure 1A–E).

### 3.2. 16S rRNA Sequencing Analysis

**Figure 2:**
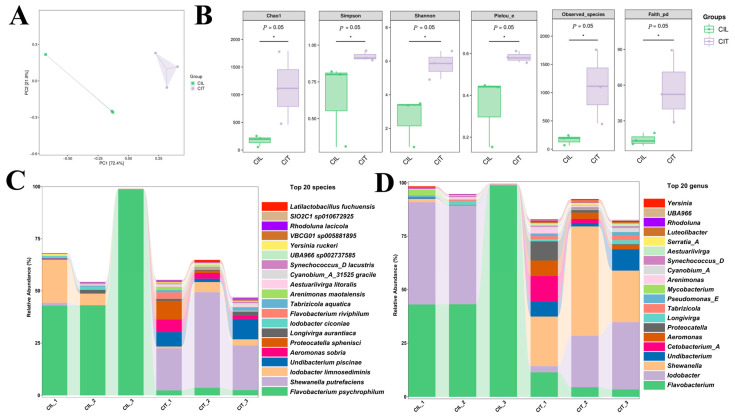
Comparison of the relative abundances of dominant bacteria in the ulcerated wounds and visceral tissues of diseased grass carp in Honghu City. CIL represents the ulcerated wound of diseased grass carp, and CIT represents the visceral tissue of diseased grass carp. (**A**) Principal Component Analysis (PCA) Plot; (**B**) Microbial community α—diversity index; (* Statistically significant difference, *p* < 0.05.) (**C**) Relative abundances (%) of bacterial taxa at the species level; (**D**) Relative abundances (%) of bacterial taxa at the genus level.

A total of 414,557 effective sequences were obtained from ulcerative wounds and visceral tissues by 16S rRNA gene sequencing after quality control and denoising. The Principal Component Analysis (PCoA) plot shows that samples from the ulcerated wounds of diseased fish (CIL group) and the visceral tissues of diseased fish (CIT group) cluster in different regions, reflecting differences in microbial community structures (Figure 2A). Significant enrichment of *Flavobacterium psychrophilum* was detected in the ulcerated tissues of diseased fish (Figure 2C,D). According to the boxplot comparison, the microbial community in the diseased fish tissues (CIT group) exhibited significantly higher species richness, diversity, and phylogenetic diversity compared to that in the ulcerated wounds of diseased fish (CIL group) (Figure 2B).

### 3.3. Morphological and Physiological-Biochemical Analysis of Hbh25210 Strain

**Figure 3:**
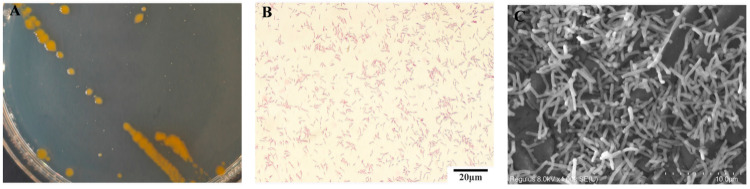
Morphological characteristics of Hbh25210 strain. (**A**) Bacterial colonies (TYES). (**B**) Gram staining (scale bar: 20 μm). (**C**) Scanning electron microscopy results (scale bar: 10 μm).

A dominant bacterial strain (designated as Hbh25210) was isolated from ulcerative lesions on the body surface of diseased fish through direct plating methodology. The isolate demonstrated slow growth on TYES agar, forming circular, convex-centered colonies with smooth mucoid surfaces and distinctive yellow pigmentation (Figure 3A). Gram-staining confirmed its classification as a Gram-negative bacterium (Figure 3B). Scanning electron microscopy revealed elongated rod-shaped cells (3~10 μm in length) lacking flagellar structures (Figure 3C). Morphological observation of single colonies, Gram staining, and scanning electron microscopy (SEM) analysis revealed that its biological characteristics were largely congruent with those of *Flavobacterium psychrophilum*.

Physiological and biochemical identification of strain Hbh25210 revealed consistent phenotypic traits with the reference strain *Flavobacterium psychrophilum*. The isolate was positive for oxidase, catalase, gelatin hydrolysis, and casein hydrolysis while being negative for starch hydrolysis, gluzyme, fructose utilization, galactose utilization, oxidation–fermentation (O/F) test, esculin hydrolysis, nitrate reduction, hydrogen sulfide (H_2_S) production, methyl red (M.R) test, Voges-Proskauer (V-P) test, ONPG test, and indole test (Table 3).

### 3.4. 16S rRNA Sequence Analysis of Hbh25210 Strain

**Figure 4:**
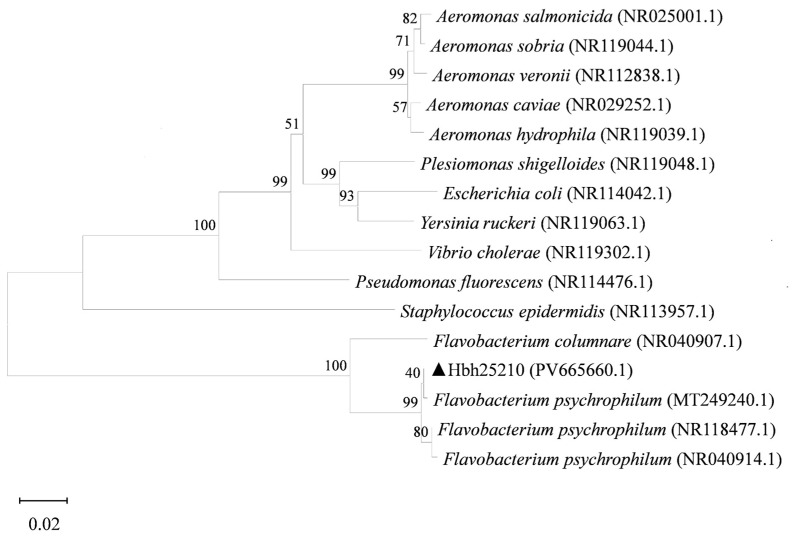
Phylogenetic tree of Hbh25210 strain based on 16S rRNA gene sequences. The phylogenetic tree was constructed using the Neighbor-Joining method in MEGA 7.0 software, incorporating sequences from representative strains of related taxa. Bootstrap values (expressed as percentages) based on 2000 replicates are shown at the nodes. The scale bar indicates the number of nucleotide substitutions per site. The black triangle indicates the experimental strain Hbh25210 in this study.

A 1349 bp sequence was amplified using the universal primers 27F and 1492R. Blastn alignment revealed that the 16S rRNA sequence of this strain exhibited 100% similarity to the 16S rRNA sequence of *Flavobacterium psychrophilum* in the NCBI database (GenBank accession NO. CP081494.1). A phylogenetic analysis revealed that the Hbh25210 strain (PV665660.1) and *Flavobacterium psychrophilum* (MT249240.1) clustered into the same clade (Figure 4).

### 3.5. Antimicrobial Susceptibility Test

The strain exhibited high-level resistance to multiple antibiotics, including ciprofloxacin, cefadroxil, neomycin, florfenicol, sulfamethoxazole, doxycycline, trimethoprim–sulfamethoxazole, erythromycin and flumequine. Conversely, it was relatively sensitive to enrofloxacin (19 mm), gentamicin (20 mm), and amikacin (22 mm) (Table 4).

### 3.6. Serotyping and Genotyping of Hbh25210 Strain

**Figure 5:**
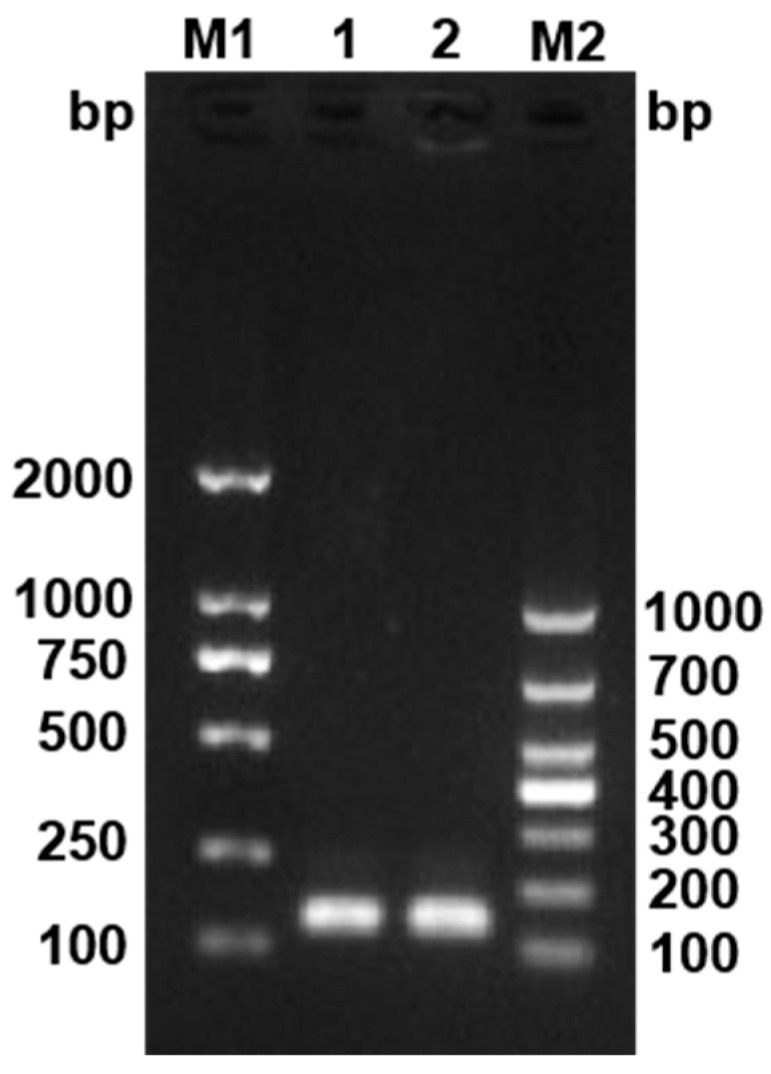
Serotyping results of Hbh25210 strain by mPCR. M1: DNA marker 2000; M2: DNA marker 1000; 1 and 2: Hbh25210 strain.

Multilocus sequence typing (MLST) analysis of strain Hbh25210 revealed the following allelic profiles for seven housekeeping genes: *trpB* (allele 5), *gyrB* (allele 24), *dnaK* (allele 8), *fumC* (allele 6), *murG* (allele 8), *tuf* (allele 1), and *atpA* (allele 8), corresponding to sequence type-51 (Table 5). Phylogenetic clustering assigned this strain to CC-ST56. Serotyping via mPCR yielded a 188 bp amplicon, consistent with serotype 0, as defined by the absence of specific somatic antigen markers. These data align with genomic surveillance frameworks for strain classification and epidemiological tracking (Figure 5).

### 3.7. Artificial Infective Experiment

**Figure 6:**
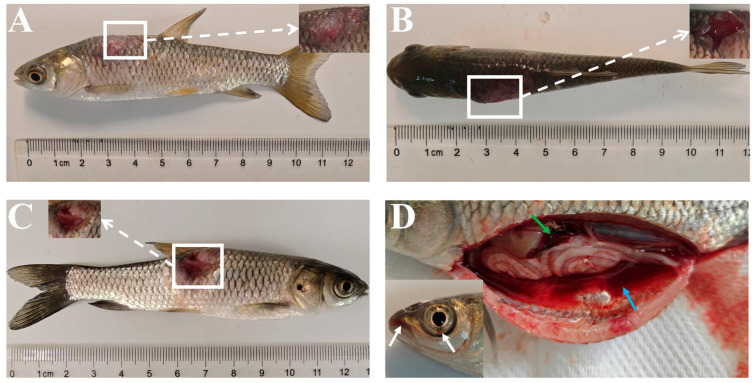
Pathogenicity observation of grass carp following intramuscular injection of strains. (**A**) Clinical signs of grass carp at 3 dpi during challenge infection; (**B**) Clinical signs at 4 dpi; (**C**) Clinical signs at 5 dpi; (**D**) Clinical and pathological dissection signs at 5 dpi, ascites (blue arrow), splenomegaly (green arrow), and hyperemia in the snout and eyeballs (white arrow).

In experimental infection trials using different concentrations of *F. psychrophilum*, mortality occurred in all treatment groups with highly consistent symptom progression across the three concentrations. At 3 days post-infection (dpi), scale loss and mild hyperemia were observed at the injection site (Figure 6A). By 4 dpi, the injection site exhibited marked swelling and protrusion (Figure 6B). At 5 dpi, muscle tissue erosion and complete exposure of blood vessels occurred at the injection site, eventually resulting in mortality of the experimental fish (Figure 6C). Necropsy revealed ascites and congestive splenomegaly, as well as marked hyperemia in the snout and eyeballs of some grass carp (Figure 6D). *Flavobacterium psychrophilum* was successfully re-isolated from lesion sites, fulfilling Koch’s postulates for pathogenicity confirmation.

**Figure 7:**
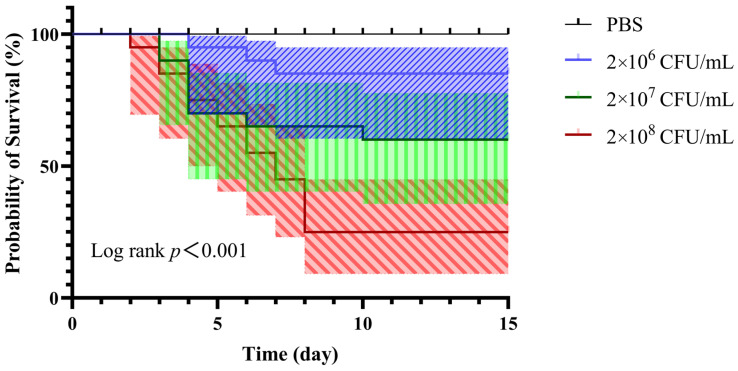
Kaplan–Meier survival curves of grass carp challenged intramuscularly with different doses of Hbh25210 strain (log-rank test, *p* < 0.001). The purple slashed region indicates the predicted mortality probability at 2 × 10^6^ CFU/mL; the green vertical lined region indicates the predicted mortality probability at 2 × 10^7^ CFU/mL; the red slashed region indicates the predicted mortality probability at 2 × 10^8^ CFU/mL.

Results of the Kaplan–Meier survival analysis demonstrated highly significant differences in survival rates among the different challenge groups (log-rank *p* < 0.001). Notably, the treatment group infected with 2 × 10^8^ CFU/mL showed persistent mortality from 2~8 dpi, with a cumulative mortality rate of 75%. The 2 × 10^7^ CFU/mL group exhibited a marked mortality peak at 4 dpi, with a mortality rate of 40%, while the 2 × 10^6^ CFU/mL group showed the lowest mortality rate of only 15% (Figure 7 and Table 6). In contrast, the control group displayed only transient local swelling at the injection site, which resolved within 2 days, with no histopathological abnormalities detected and zero mortality throughout the observation period.

### 3.8. Histopathological Analysis

**Figure 8:**
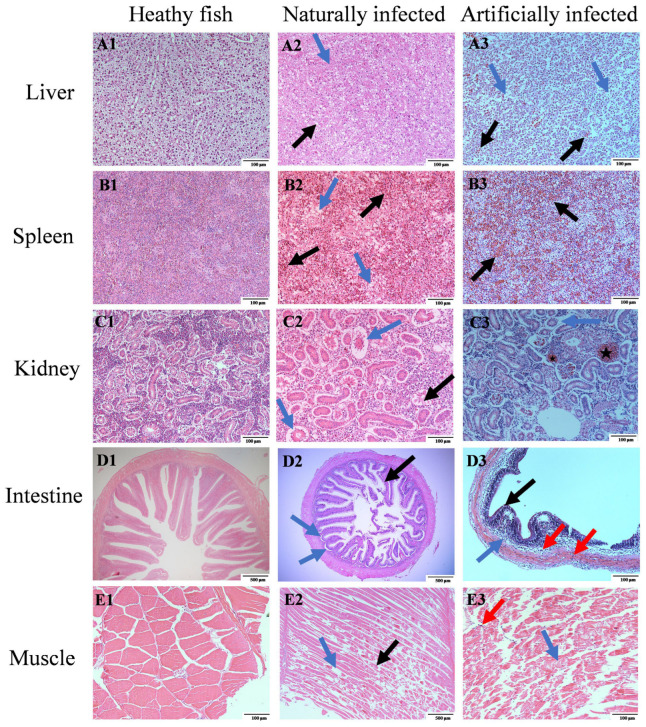
Pathological observation: control group and *Flavobacterium psychrophilum* infected grass carp. Tissue sections were stained with hematoxylin and eosin (H&E staining). (**A1**–**E1**) Tissue sections of liver, spleen, kidney, intestine, and muscle in the control group. (**A2**) In diseased fish liver, disappearance of cell nuclei (blue arrow) and vacuoles of fatty degeneration (black arrow). (**A3**) Massive necrosis of hepatocytes (blue arrow) and bleeding due to blood vessel rupture (black arrow) in diseased fish liver. (**B2**) Necrotic vacuoles (blue arrow) and massive congestion (black arrow) in diseased fish spleen cells. (**B3**) Congestion in diseased fish spleen (black arrow). (**C2**) Swelling of renal tubules and glomeruli (blue arrow), rupture of some renal tubular epithelial cells (black arrow) in diseased fish kidney. (**C3**) Swelling of renal tubules and glomeruli (blue arrow), congestion of blood vessels in the kidney (★) in diseased fish. (**D2**) Obvious gaps in the submucosa of diseased fish intestine (blue arrow), rupture of goblet cells (black arrow). (**D3**) Enlarged submucosal spaces (blue arrow) and inflammatory cell infiltration in both submucosa and circular muscle (red arrow), near disappearance of goblet cells (black arrow) in diseased fish. (**E2**) Muscle fiber breakage (blue arrow) and sarcoplasm dissolution (black arrow) in diseased fish muscle. (**E3**) Bent muscle fibers (blue arrow) accompanied by lymphocyte infiltration (red arrow) in diseased fish muscle.

Histopathological examination was performed on tissue samples from farmed diseased fish collected in Honghu City, experimentally infected grass carp at 72 hpi, and healthy grass carp controls. Healthy grass carp hepatocytes showed a tight regular arrangement with narrow sinusoids (Figure 8A1). In contrast, liver tissue from Honghu City diseased fish showed loss of hepatocyte boundaries, nuclear disappearance, intracellular fluid leakage, and vacuolation (Figure 8A2). Experimentally infected fish displayed hepatocyte necrosis and vascular hemorrhage (Figure 8A3).

Healthy grass carp spleen presented distinct boundaries between red pulp and white pulp (Figure 8B1). Honghu City diseased fish showed splenocyte necrosis, accumulation of red blood cells, and blurred splenic red pulp–white pulp boundaries (Figure 8B2). Experimentally infected fish showed similar boundary blurring and red blood cell accumulation in splenocytes (Figure 8B3).

Healthy grass carp renal cells were compactly arranged with a regular structural morphology (Figure 8C1). Honghu City diseased fish had swollen renal tubules and glomeruli, along with ruptured renal tubular epithelial cells (Figure 8C2). Experimentally infected fish exhibited swelling of renal tubules and glomeruli and renal vascular congestion (Figure 8C3).

Healthy grass carp intestine showed an intact mucosal epithelium, regular intestinal villi, and ordered smooth muscle layers (Figure 8D1). Honghu City diseased fish had obvious submucosal gaps and ruptured goblet cells (Figure 8D2). Experimentally infected fish presented enlarged submucosal spaces, inflammatory cell infiltration in both submucosa and circular muscle layers, and nearly absent goblet cells (Figure 8D3).

Healthy grass carp muscle fibers showed a regular arrangement without an inflammatory response (Figure 8E1). Honghu City diseased fish had fractured muscle fibers with sarcoplasmic lysis (Figure 8E2). Experimentally infected fish showed curved muscle fibers accompanied by lymphocyte infiltration (Figure 8E3).

### 3.9. Tissue Tropism of Flavobacterium psychrophilum in Infected Grass Carp

**Figure 9:**
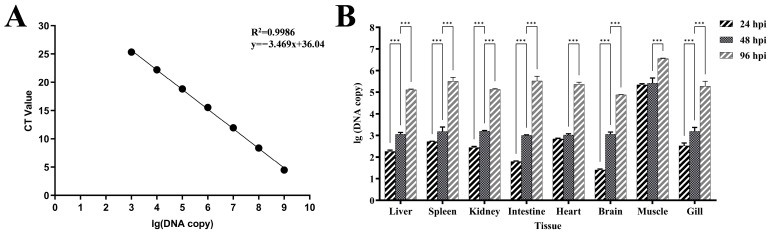
RT-qPCR-Based Quantification: Standard Curve and Tissue Tropism of *Flavobacterium psychrophilum* in Grass Carp. (**A**) Standard curve established based on the *rpoC* fragment of Hbh25210 strain; (**B**) Tissue distribution dynamics of Hbh25210 strain after intramuscular infection in Grass Carp. *** *p* < 0.001.

Taking the lg value of the *rpoC* gene copy number in DNA as the x-axis and the CT value of qPCR as the y-axis, the equation of the standard curve was fitted as y = −3.469x + 36.04 (slope −3.469, intercept 36.04). The correlation coefficient (R^2^ = 0.9986) indicated an excellent linear relationship (Figure 9A).

Quantitative analysis using TaqMan qPCR—targeting the *rpoC* gene—revealed the spatiotemporal dynamics of the *Flavobacterium psychrophilum* distribution across multiple tissues of grass carp throughout the infection course. At 24 hpi, the infected muscle showed the highest bacterial load of 5.07 × 10^5^ copies /μL, followed by the heart at 1.60 × 10^3^ copies/μL and the spleen at 1.20 × 10^3^ copies/μL. At 48 hpi, the bacterial load in infected muscle increased to 5.96 × 10^5^ copies/μL, with the kidney, gill filament and spleen showing 3.60 × 10^3^, 3.55 × 10^3^ and 3.47 × 10^3^ copies/μL, respectively, while other tissues maintained levels around 10^3^ copies/μL. At 96 hpi, the ulcerated muscle reached a peak bacterial load of 8.13 × 10^6^ copies/μL, followed by the intestine at 7.55 × 10^5^ copies/μL, the spleen at 7.23 × 10^5^ copies/μL and the heart at 5.27 × 10^5^ copies/μL, with other tissues stabilized at 10^5^ copies/μL (Figure 9B).

Time-course analysis showed significant proliferation of bacterial loads in all tissues at 96 hpi compared to 48 hpi. The intestine exhibited the highest proliferation rate (325.81-fold), followed by the heart (218.17-fold), spleen (208.15-fold), gill (121.83-fold), liver (114.62-fold), kidney (86.22-fold), brain (66.06-fold) and muscle (13.64-fold). The infected muscle maintained the highest bacterial load at all time points examined.

## 4. Discussion

*Flavobacterium psychrophilum* is a psychrophilic pathogen and the primary cause of bacterial coldwater disease and rainbow trout fry syndrome in salmonids, which has severely restricted salmonid aquaculture [13,14]. The bacterium typically causes outbreaks at 6–10 °C [15]. It colonizes and degrades fish tissues via proteases and adhesins, using host proteins for growth. It adapts to microaerobic conditions through facultative anaerobism and oxidative stress systems, and persists in water via biofilms and cyanophycin. These metabolic adaptations and tissue affinity synergize to drive its global spread in salmonid aquaculture [16]. Current studies primarily focus on rainbow trout and salmonid fish, with mortality rates as high as 90% in rainbow trout fry [17,18]. Infected adult fish typically exhibit anorexia, splenitis, extensive skin lesions, and ulcerative necrosis of skeletal muscle, with inflammatory infiltration and cellular damage to the spleen, kidney, intestinal wall and other tissues [19,20,21,22]. However, as the country with the highest freshwater aquaculture output in the world, China has few reports on the overwintering mortality syndrome of major freshwater fish species.

In this study, *Flavobacterium psychrophilum* was detected in overwintering mortality-diseased grass carp exhibiting congestion, cutaneous lesions, focal muscle ulcers, and occasional hepatic abnormalities. 16S rRNA sequencing showed significantly lower microbial richness and evenness in ulcerated muscles than viscera, with *Flavobacterium psychrophilum* and *Iodobacter limnosediminis* being dominant—*Flavobacterium psychrophilum* at 98.80% in the ulcerated wounds of diseased fish. Pathogenicity was confirmed through experimental challenge, with clinical manifestations consistent with those observed in naturally infected fish. *Iodobacter limnosediminis*, weakly pathogenic to *Salmo trutta* and coexisting with *Flavobacterium psychrophilum* at lesions, may synergize in pathogenesis [23]. Besides *Flavobacterium psychrophilum*, visceral tissues harbor other psychrophilic pathogens (e.g., *Yersinia ruckeri*) and weak pathogens (e.g., *Aeromonas sobria*, *Shewanella putrefaciens*). Notably, opportunistic *Shewanella putrefaciens* shows adhesion–spoilage correlation; high-adhesion strains increase intestinal *Parabacteroides* and macrophages, inducing the production of volatiles and exacerbating fatty acid oxidation. It causes skin ulcers and visceral damage at low temperatures via biofilm and virulence gene expression [24,25]. In the scenario of polymicrobial infection as described above, various pathogens secrete large quantities of exotoxins and toxic metabolites. These factors synergistically damage host tissues, interfere with immune signaling, severely compromise the immune defense capacity of the fish, and exacerbate the onset and progression of overwintering disease. Based on previous studies, we suggest that a low temperature reduces the metabolic rate and immunity of fish, which impairs the clearance of opportunistic pathogens under cold conditions, thereby leading to a shift in the microbial community from a symbiotic state to a pathogenic state [6,26,27,28].

*Flavobacterium psychrophilum* has an optimal growth temperature of 4–20 °C and can adapt to low-temperature environments by regulating gene expression through temperature sensing, thereby efficiently colonizing and infecting grass carp during overwintering [29]. Previous studies have shown that a low temperature significantly upregulates the expression of virulence genes such as *Fpp1*, which in turn causes typical symptoms including scale loss and muscle ulceration [30]. In summary, a low temperature provides suitable growth conditions for the bacterium, leading to marked opportunistic pathogenicity in grass carp with weakened immunity during overwintering and exacerbating morbidity and mortality. High expression of these extracellular metalloproteases induces typical symptoms in diseased grass carp in this study, including scale loss and muscle ulceration. *Flavobacterium psychrophilum* virulence activation varies with infection route: intramuscular injection better reveals virulence gene effects than intraperitoneal or oral injection [31]. For example, 1.3–5.6 g rainbow trout had the highest mortality with typical symptoms after a 15 °C bacterial bath [32]. Although immersion infection is more consistent with natural transmission routes (which require breaching mucosal barriers), this method is affected by complex aquatic environments, leading to large fluctuations in mortality. Our previous epidemiological surveys also revealed that most diseased grass carp suffered mechanical injuries before overwintering, such as those caused by seining, pond transfer and other farming operations. These wounds healed slowly at low temperatures, leading to impaired mucosal barrier integrity and significantly increased host susceptibility to *Flavobacterium psychrophilum*, facilitating pathogen invasion through lesions. This natural invasion process also bypasses the mucosal immune barrier, similar to the intramuscular challenge model used in this study. For these reasons, intramuscular injection was adopted as the infection route. However, the artificial infection route differs from natural infection pathways, which represents a major limitation of this study.

Compared with naturally diseased fish, grass carp subjected to artificial challenge infection showed no green liver within 15 days. Relevant studies on yellow catfish have suggested that excessive intake of feed rich in plant ingredients may lead to high dietary pectin content, which in turn interferes with bile acid synthesis and excretion and induces intrahepatic cholestasis. This may represent a potential cause underlying the occurrence of green liver in grass carp [33]. Grass carp reovirus was detected by nested PCR (Table S1 and Figure S1). This virus latently infects brain tissue at low temperatures and may reduce host immunity via chronic inflammation, thereby increasing susceptibility of overwintering grass carp to pathogens such as *Flavobacterium psychrophilum* and *Shewanella putrefaciens* [34]. Tissue tropism analysis showed that bacterial loads were relatively high in the gill, spleen, heart and muscle at 24 hpi. We hypothesize that intramuscular injection induces skeletal muscle apoptosis, and that the dorsal fin is adjacent to spinal vessels, enabling bacteria to enter the blood circulation and preferentially colonize blood cell-rich tissues including the gill, heart and spleen [21]. At 48 hpi, bacterial loads in all tissues tended to be consistent except for in muscle. This may be attributed to the activation of the host innate immune system following pathogen invasion of immune-related organs, where key factors including IgM, complement component C3, and lysozyme participate in eliminating exogenous pathogens, thereby balancing bacterial loads across tissues [35]. At 96 hpi, bacterial loads in all examined tissues reached the highest level. In the present study, we found that bacterial loads in the intestine and brain of grass carp showed extremely significant increases at all time points during bacterial proliferation. The rich microbial community in the intestine plays an effective role against *Flavobacterium psychrophilum* at the early stage of infection (24 hpi). Moreover, studies in rainbow trout have demonstrated that the intestine can maintain a high level of ROS for a longer period than other tissues to eliminate pathogens during infection with *F. psychrophilum* [36]. Previous studies have shown that fish can resist bacterial invasion into brain tissue through the synergistic action of the blood–brain barrier, medulla oblongata immune region and immune tolerance, which may be an important mechanism for their early resistance to *Flavobacterium psychrophilum* infection [37].

Pathologically, spleen (necrosis and congestion), kidney (renal tubule enlargement and congestion), and muscle (myofiber rupture with lymphocyte infiltration) lesions resemble those in rainbow trout [38,39]. Liver lesions such as hepatocellular necrosis and hemorrhage were consistent in naturally and experimentally infected fish, while intestinal lesions differed significantly: natural infection resulted in mild injury, whereas experimental infection caused intestinal villi shedding and goblet cell loss, which may be related to mixed bacterial infection or fish size. No such intestinal pathological changes have been reported in salmonids. Recent epidemiological investigations by our group showed that pre-wintering operations such as seining and pond transfer easily cause scale loss and epidermal damage in grass carp, providing an entry route for low-temperature pathogens. When the water temperature stabilizes at approximately 10 °C, *Flavobacterium psychrophilum* proliferates massively at the wounds and induces tissue ulceration, leading to an increase in the saprophytic bacterium *Shewanella putrefaciens*, and ultimately results in overwintering mortality syndrome.

MLST technology enables accurate classification and epidemiological tracing of bacteria through standardized typing, whereas serotyping provides a scientific basis for the diagnosis and prevention of related diseases. Currently, the widely used serotyping scheme for *Flavobacterium psychrophilum* classifies the bacterium into four serotypes: 0, 1, 2, and 3 [5]. The *Flavobacterium psychrophilum* strain Hbh25210 in this study belongs to CC-ST56 (including ST-51). Only 3 strains assigned to this clonal complex are currently recorded in the MLST database, all from Japanese cyprinids: crucian carp, *Zacco platypus*, and common carp [4]. Previous studies have shown that the grass carp-derived strain Ci2402 (ST-51) reported by Zhang et al. and the crucian carp-derived strain NJ01 (ST-363) reported by Jiang et al. both belong to CC-ST56, indicating a close genetic relationship among *Flavobacterium psychrophilum* isolates from cyprinids in China [38,40]. The *Flavobacterium psychrophilum* isolate in this study was identified as serotype 0, which is consistent with that from *Oncorhynchus masou* in China. According to Li et al., most *F. psychrophilum* isolates belonged to serotypes 0 and 1, and a strong association was observed between serotype and host fish species [41]. Although serotype 0 exhibited lower prevalence and virulence in rainbow trout compared with serotypes 1 and 2, it showed high pathogenicity to grass carp in the present study, suggesting that serotype 0 may pose a high infection and pathogenic risk to cyprinids such as grass carp [42,43].

Control of *Flavobacterium psychrophilum* infections faces multiple challenges: unclear pathogenic mechanisms, prominent antigenic diversity, and inter-strain variation limit the efficacy of inactivated vaccines and constrain the application of attenuated live vaccines, while delayed progress in understanding fish immunity and antigenic protection has slowed vaccine development [14,44]. Although immersion vaccination with live bacteria confers superior immune protection in rainbow trout fry compared to attenuated or inactivated vaccines, wild-type live strains pose substantial environmental contamination risks under field conditions [31], and existing inactivated multivalent vaccines lack rigorous protective efficacy evaluation in rainbow trout [45]. Limited vaccine availability has promoted increased antibiotic usage, exacerbating the spread of resistance genes. Notably, no standardized antimicrobial breakpoints exist for *Flavobacterium psychrophilum*, so susceptibility descriptions below are based on inhibition zones. The strain characterized in this study demonstrated relative susceptibility only to enrofloxacin, gentamicin, and amikacin while exhibiting resistance to erythromycin, cefadroxil, neomycin, and other antimicrobials. However, the antimicrobial susceptibility test in this study was performed without quality control strains and biological replicates. These methodological limitations may affect the reliability and repeatability of the results, which should be regarded as preliminary and for reference only. This resistance profile differs markedly from most previously characterized strains, including florfenicol-sensitive Slovenian isolates and Canadian strains relatively susceptible only to erythromycin, and likely reflects selective pressure from continuous or frequent antimicrobial switching in local farms during early mortality events, giving rise to its multidrug-resistant phenotype. *Flavobacterium psychrophilum* isolated from *Carassius auratus gibelio* in Jiangsu Province, China, was gentamicin-sensitive, consistent with our findings [38]. Antimicrobial susceptibility profiles of isolates from other regions differ entirely. For example, Chilean salmon farm strains are florfenicol-sensitive but exhibit reduced susceptibility to oxytetracycline and certain quinolones [46]; Great Lakes strains from Michigan are fully susceptible to florfenicol, with 76% being oxytetracycline-sensitive [8]; United Kingdom isolates are fully florfenicol-susceptible, with most showing reduced susceptibility to oxolinic acid and oxytetracycline [47]; Slovenian strains are florfenicol- and erythromycin-sensitive, with over 86.3% exhibiting high-level resistance to oxolinic acid, oxytetracycline, and enrofloxacin [48]; Canadian strains show prominent sulfonamide combination resistance, with >50% being resistant to florfenicol and oxytetracycline while remaining relatively susceptible only to erythromycin [29]. In summary, the Hbh25210 strain was resistant to 9 common antibiotics, a pattern closely related to local antimicrobial usage practices. Prolonged and inappropriate drug use has led to the emergence of multidrug-resistant strains, leaving few effective treatments for disease outbreaks, while horizontal transmission of resistance genes can induce resistance in more bacterial species. Given the current lack of effective vaccines, priority measures include selecting drugs based on antimicrobial susceptibility test results, standardizing dosages and treatment courses, reducing indiscriminate use of broad-spectrum antimicrobials, and accelerating the development of novel vaccines.

This study has certain limitations. Owing to constraints in experimental facilities and water temperature control, we were unable to fully reproduce the natural infection model of grass carp, and only performed challenge trials using juvenile individuals, focusing solely on pathogenic microorganisms to observe the etiology of overwintering mortality syndrome. Furthermore, overwintering mortality syndrome is a multifactorial disorder shaped by multiple contributors, including gill microbial dysregulation-induced ferroptosis [49], increased pathogen susceptibility, and impaired intestinal barrier during overwintering [50]. Nevertheless, the findings of this study sufficiently confirm that *Flavobacterium psychrophilum* is pathogenic to grass carp and is an important pathogen associated with overwintering mortality syndrome [51].

## 5. Conclusions

*Flavobacterium psychrophilum* strain Hbh25210 was isolated from ulcerative lesions on the body surface of grass carp affected by overwintering mortality syndrome and was highly enriched in these lesions. Experimental infection confirmed its pathogenic potential, reproducing natural symptoms and causing 15–75% mortality. The strain was characterized as serotype 0 and sequence type ST-51 (CC-ST56). Bacterial loads were highest in muscle, followed by the intestine and spleen, peaking at 96 h post-infection. Antimicrobial susceptibility testing revealed multidrug resistance, though the strain remained relatively sensitive to enrofloxacin, gentamicin, and amikacin. These findings demonstrate that *Flavobacterium psychrophilum* is an important pathogen associated with grass carp overwintering mortality and provide essential insights for diagnosis and targeted control strategies. Further research is needed to explore the pathogenic mechanism of *Flavobacterium psychrophilum* and develop effective prevention and control measures in aquaculture practice.

## Figures and Tables

**Table 1:** Primers used in this study.

Primer Name	Sequence (from 5′ to 3′)	Band Size (bp)	Reference
341F	F: CCTACGGGNGGCWGCAG	466	[3]
806R	R: GGACTACHVGGGTATCTAAT		
27F	F: AGAGTTTGATCCTGGCTCAG	1354	[3]
1492R	R: TACGGCTACCTTGTTACGACTT		
*trpB*	F: CAGGAAACAGCTATGACCAAGATTATGTAGGCCGCCC	789	[4]
	R: TGTAAAACGACGGCCAGTTGATAGATTGATGACTACAATATC		
*gyrB*	F: CAGGAAACAGCTATGACCGTTGTAATGACTAAAATTGGTG	1077	[4]
	R: TGTAAAACGACGGCCAGTCAATATCGGCATCACACAT		
*dnaK*	F: CAGGAAACAGCTATGACCAAGGTGGAGAAATTAAAGTAGG	873	[4]
	R: TGTAAAACGACGGCCAGTCCACCCATAGTTTCGATACC		
*fumC*	F: CAGGAAACAGCTATGACCCCAGCAAACAAATACTGGGG	750	[4]
	R: TGTAAAACGACGGCCAGTGGTTTACTTTTCCTGGCATGAT		
*murG*	F: CAGGAAACAGCTATGACCTGGCGGTACAGGAGGACATAT	681	[4]
	R: TGTAAAACGACGGCCAGTGCATTCTTGGTTTGATGGTCTTC		
*tuf*	F: CAGGAAACAGCTATGACCGAAGAAAAAGAAAGAGGTATTAC	795	[4]
	R: TGTAAAACGACGGCCAGTCACCTTCACGGATAGCGAA		
*atpA*	F: CAGGAAACAGCTATGACCCTTGAAGAAGATAATGTGGG	834	[4]
	R: TGTAAAACGACGGCCAGTTGTTCCAGCTACTTTTTTCAT		
Ctrol	F: AGCAAATTTGGCTCTTTTGG	188	[5]
	R: TTGTAACAACGCCACCAGTT		
Type-1	F: ACCAACCTTCAAGATTATCGT	549	[5]
	R: GGGGAGTGGTTAGAACTGA		
Type-2	F: TTGAACGAAACTTATATGGATAGA	841	[5]
	R: TTACCAAAGAGCCCTTTAGTG		
Type-3	F: CGCCATGCAAGAAATTAGTT	361	[5]
	R: CCTGCGATCTCAACATATCA		

**Table 2:** Gene-specific primers and TaqMan probe for *rpoC* amplification.

Primer Name	Sequence (from 5′ to 3′)	Reference
*rpoC*-F	GAAGATGGAGAAGGTAATTTAGTTGATATT	[12]
*rpoC*-R	CAAATAACATCTCCTTTTTCTACAACTTGA	
Taqman probe	FAM-AAACGGGTATTCTTCTTGCTACA-BHQ1	

**Table 3:** Physiological and biochemical identification of Hbh25210 strain.

Reaction Item	Hbh25210 Result	*F. psychrophilum* Result
Oxidase	+	+
Catalase	+	+
Gelatin hydrolysis	+	+
Casein hydrolysis	+	+
Starch hydrolysis	-	-
Gluzyme	-	-
Fructose	-	-
Galactose	-	-
O/F	-	-
Esculin hydrolysis	-	-
Nitrate reduction	-	-
H_2_S production	-	-
M.R test	-	-
V-P test	-	-
ONPG	-	-
Indole	-	-

Notes: “+” means positive reaction; “-” means negative reaction.

**Table 4:** Detection of drug sensitivity of the Hbh25210 strain.

Antibiotics	Content (μg·disc^−1^)	Inhibition Zone/mm
Ciprofloxacin	5	0
Cefadroxil	30	0
Neomycin	30	0
Enrofloxacin	5	19
Florfenicol	30	0
Gentamicin	10	20
Amikacin	30	22
Sulfamethoxazole	300	0
Doxycycline	30	0
Trimethoprim–Sulfamethoxazole	25	0
Erythromycin	15	0
Flumequine	30	0

**Table 5:** MLST typing of isolate Hbh25210 based on multilocus sequence analysis.

Isolation	Allele Type							Genotype	Clonal Complex
	*trpB*	*gyrB*	*dnaK*	*fumC*	*murG*	*tuf*	*atpA*		
Hbh25210	5	24	8	6	8	1	8	ST-51	CC-ST56

**Table 6:** Cumulative Mortality of Grass Carp Following *Flavobacterium psychrophilum* Challenge.

Treatment	Day Post-Infection (dpi)														
	1	2	3	4	5	6	7	8	9	10	11	12	13	14	15
PBS	0	0	0	0	0	0	0	0	0	0	0	0	0	0	0
2 × 10^6^ CFU/mL	0	0	0	1	0	1	1	0	0	0	0	0	0	0	0
2 × 10^7^ CFU/mL	0	0	2	4	0	1	0	0	0	1	0	0	0	0	0
2 × 10^8^ CFU/mL	0	1	2	2	2	2	2	4	0	0	0	0	0	0	0

## Data Availability

Data will be made available upon reasonable request.

## Supplementary Materials

The following supporting information can be downloaded at: https://www.mdpi.com/article/10.3390/ani16101465/s1, Table S1. Primers used in this study. Figure S1. Nested PCR gel electrophoresis image for the detection of GCRV-II (second round). M: Marker; 1: Negative control; 2: Positive control; 3–4: Test samples.
