# Supplementary material for: Flavobacterium psychrophilum as an Important Pathogen Associated with Overwintering Mortality Syndrome in Grass Carp (Ctenopharyngodon idella): Isolation, Characterization, and Pathogenicity
Source: Animals (Basel). 2026 May 10;16(10):1465. doi: 10.3390/ani16101465 (PMC13203417; doi:10.3390/ani16101465)
Supplement: Supplementary file 1 [file animals-16-01465-s001.zip › animals-4250030-supplementary.pdf]

Supplementary Materials

Total RNA was extracted from tissue of naturally infected grass carp using the TRIzol method and reverse-transcribed to synthesize cDNA. Nested PCR was performed to detect the presence of GCRV-II in fish, following the Technical Specification for Surveillance of Grass Carp Hemorrhagic Disease SC/T 7023-2021 (National public service platform). The specific primers used are listed in Table S1. Gel electrophoresis revealed an amplicon of approximately 363 bp, confirming GCRV infection in the naturally diseased grass carp (Figure S1).

Table S1. Primers used in this study.

Primer Name	Sequence (from 5′ to 3′)	Band Size (bp)	Reference
GCRV-II-SP	CGCGATTTCATACCCTTTCT	408	[51]
GCRV-II-OUTP	TAGCTGCCCTACTTGGGATGA		
GCRV-II-INP	CATACGATCGCTCCCAACTCC	363	
GCRV-II-SP	CGCGATTTCATACCCTTTCT		

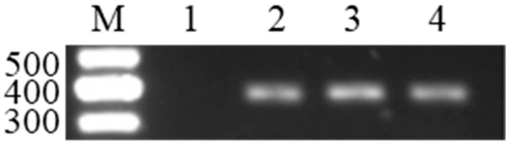

Figure S1. Nested PCR gel electrophoresis image for the detection of GCRV-II (second round). M: Marker; 1: Negative control; 2: Positive control; 3–4: Test samples.

References:

51. Xu, C.; Yang, J.; Cao, J.; Jiang, N.; Zhou, Y.; Zeng, L.; Zhong, Q.; Fan, Y. The Quantitative Proteomic Analysis of Rare Minnow, *Gobiocypris rarus,* Infected with Virulent and Attenuated Isolates of Grass Carp Reovirus Genotype II. *Fish Shellfish Immunol.* **2022**, *123*, 142–151. https://doi.org/10.1016/j.fsi.2022.02.037.
